# Breast Reconstruction with a Tissue Engineering and Regenerative Medicine Approach (Systematic Review)

**DOI:** 10.1007/s10439-019-02373-3

**Published:** 2019-10-01

**Authors:** E. Donnely, M. Griffin, P. E. Butler

**Affiliations:** 1 Division of Surgery and Interventional Science, University College London, Royal Free Hospital Campus, London, UK; 2 Department of Plastic Surgery, Royal Free Hospital, London, UK

**Keywords:** Breast reconstruction, Tissue engineering, Regenerative medicine, Scaffold, Cellular therapy, Stem cells, Adipose-derived stem cells

## Abstract

Current techniques for breast reconstruction include an autologous-tissue flap or an implant-based procedure, although both can impose further morbidity. This systematic review aims to explore the existing literature on breast reconstruction using a tissue engineering approach; conducted with the databases Medline and Embase. A total of 28 articles were included, mainly comprising of level-5 evidence with *in vitro* and animal studies focusing on utilizing scaffolds to support the migration and growth of new tissue; scaffolds can be either biological or synthetic. Biological scaffolds were composed of collagen or a decellularized tissue matrix scaffold. Synthetic scaffolds were primarily composed of polymers with customisable designs, adjusting the internal morphology and pore size. Implanting cells, including adipose-derived stem cells, with combined use of basic fibroblast growth factor has been studied in an attempt to enhance tissue regeneration. Lately, a level-4 evidence human case series was reported; successfully regenerating 210 mL of tissue using an arterio-venous pedicled fat flap within a tissue engineering chamber implanted on the chest wall. Further research is required to evaluate whether the use of cells and other growth factors could adjust the composition of regenerated tissue and improve vascularity; the latter a major limiting factor for creating larger volumes of tissue.

## Introduction

Breast cancer poses a significant problem, being the most common cancer in the UK female population. In 2015 alone there were 54,800 new cases diagnosed, with incidence rates projected to increase 2% by the year 2035.^4^ Despite this increase, better screening and treatment options are leading to an improvement in survival rates, meaning almost eight out of 10 women survive greater than 10 years after diagnosis.^4^ By the end of 2010, there was just under half a million women in the UK living with a diagnosis of breast cancer. And its not just affecting the UK, with an estimated two million new cases diagnosed worldwide in 2018.^2^

Following a diagnosis of breast cancer, 81% of patients undergo surgery to remove the primary tumor.^4^ This can be accompanied by neoadjuvant or adjuvant chemotherapy and radiotherapy, tailored according to the individual case. Surgery, whether lumpectomy or mastectomy, has long been associated with a significant burden of disease; the disfiguring surgical procedure leading to psychological distress, loss of femininity, sexual dysfunction and even suicidal ideation.^23^,^28^

Breast reconstruction can be offered immediately at the time of initial surgery in the case of neoadjuvant therapy, or as a delayed procedure following any required adjuvant therapy and necessary planning. The timing of reconstruction is highly dependant on such therapy, with a delayed autologous tissue flap more indicative if postmastectomy radiation is required due to an increased risk of capsular contracture or flap failure if performed as an immediate procedure prior to radiation.^26^ Patients undergoing a mastectomy plus breast reconstruction have a significantly decreased incidence of anxiety and depression when compared with mastectomy alone.^6^ Breast reconstruction provides an aesthetic benefit and the current standard of practice is either an autologous tissue flap or implant based (silicon or saline) procedure. However both can impose further morbidity that should not be unheeded and require an in-depth discussion between clinician and patient evaluating the risks and benefits of each. The sequalae of an autologous tissue flap includes the short-term complication of flap failure and long-term complications of fat necrosis and donor site morbidity^17^ while the silicone/saline implant based procedure incurs short-term risk of infection and long-term risks of capsular contracture and implant rupture.^15^ Textured implants are at present under review due to a possible association with anaplastic large cell lymphoma.^3^ These risks emphasise the need for a new technique of breast reconstruction.

Since stem cells were first identified, scientists and health professionals alike have been working toward repairing and replacing human tissue damaged secondary to disease and trauma using tissue engineering and regenerative medicine. Their aim of using autologous cells to proliferate and replenish the desired tissue could eliminate the long-term complications associated with either technique, such as capsular contracture or the need to harvest an autologous flap and hence donor site morbidity.

The breast is composed of glandular and adipose tissue, the latter forming majority of the volume. Autologous fat grafting has been used to fill soft tissue defects within the body, including the breast, although the limitation of resorption and unable to maintain volume for significant periods of time still persists.^13^ Cell-assisted lipotransfer involves the enrichment of the fat graft with adipose-derived stem cells, hypothesizing the ability to withstand longer periods of hypoxia. Although one study still reported loss of 47% of the initial post-operative volume following breast augmentation.^24^,^34^ To address this issue, the idea of designing a support structure capable of providing structural integrity to the developing tissue has been hypothesized and is leading the way to a tissue engineered breast.

This article aims to explore and perform a systematic review of the current literature on breast reconstruction using a tissue engineering and regenerative medicine approach.

## Methods

A systematic review was conducted with the literature databases Medline (OvidSP; 1946 to 6/12/2018) and Embase (Ovid; 1974 to 12/12/2018), adhering to the PRISMA guidelines.

The search criteria was formulated to identify articles on ‘breast reconstruction’ AND ‘tissue engineering’. The base search filters for ‘breast reconstruction’ included {MAMMAPLASTY/OR MASTECTOMY/(MeSH terms)} OR {“breast reconstruct*” OR mamm?plasty OR mastectomy OR (breast AND lumpectomy) (keywords)}. The base search filters for ‘tissue engineering’ included {TISSUE ENGINEERING/OR BIOMEDICAL ENGINEERING/OR REGENERATIVE MEDICINE/(MeSH terms)} OR {(“tissue engineer*” OR “biomedical engineer*” OR “regenerative medicine” OR biomaterial* OR “additive manufactur*” OR “3D print*” OR scaffold) (keywords)} OR {(TISSUE SCAFFOLDS/(MeSH term) OR (“tissue scaffold*” OR scaffold*) (keywords)) AND (lipofill* OR lipotransfer* OR “fat graft*” OR “adipo* stem cell*” OR “adipo* derived stem cell*” OR ADSC) (keywords)}.

The search was restricted to the English language. Letters and review articles were excluded. The inclusion criteria incorporated the use of an implantable scaffold ± cellular therapy to engineer tissue for the purpose of breast reconstruction. Articles regarding the use of commercially available breast implants were excluded, as the aim of the review was to highlight methods that reconstruct the breast with autologous tissue only and avoid the implantation and hence complications of permanent foreign devices.

## Results

**Figure 1:**
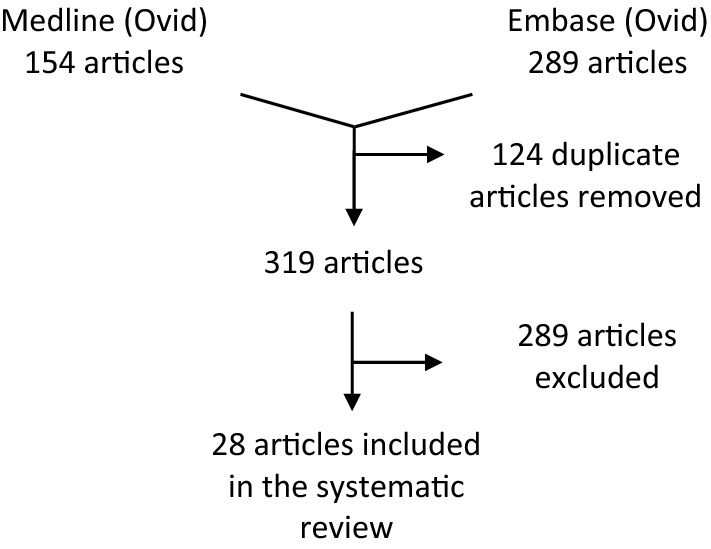
A flow chart illustrating the database search and exclusion criteria to identify the articles included.

The systematic review identified 28 articles comprising mainly of level five evidence *in vitro* and animal studies, and two level four evidence human case series (Fig. 1). Current techniques have focussed on two different biological scaffolds (eight articles; Table 1) and 13 synthetic scaffolds (fifteen articles; Table 2) to mimic the shape and support of native breast tissue. One article the scaffold material was not stated. A further four articles were identified that describe the reconstruction of the nipple areolar complex (Table 3).

**Table 1:** Biological scaffolds: systematic review data extraction

Authors	Title	Year	Journal	Study aim	Study model	Scaffold	Scaffold—biological/synthetic, bio/non-biodegradable	Volume/size of scaffold	Cell therapy	Cell niche ± growth factor
Huss and Kratz	Mammary epithelial cell and adipocyte co-culture in a 3-D matrix: the first step towards tissue-engineered human breast tissue	2001	*Cells Tissues Organs*	To culture human mammary epithelial cells with adipocytes on a 3-D biological matrix	*In vitro* model.	Collagen gel	Biological Biodegradable	Unknown—gel	Human mammary epithelial cells with adipose tissue	Dulbecco’s modified Eagles medium (DMEM) with 10% fetal calf serum (FCS) and antibiotics DMEM/Hams F12 with insulin, transferrin, triiodothyronine, hydrocortisone, cholera toxin, epithelial growth factor, 7%FCS, 1.5% adenine and antibiotics.
Tada and Fujisato	Adipose tissue engineering for a breast reconstruction (Conference abstract)	2012	*Journal of Tissue Engineering and Regenerative Medicine*	To engineer adipose tissue using decellularized matrix obtained from rat lungs	*In vivo* animal model Athymic mice	Decellularized rat lungs.	Biological Biodegradable	Unknown	Adipose cell line 3T3-L1	A circulatory culture system
Tsuji *et al.*	Simple and longstanding adipose tissue engineering in rabbits	2012	*Journal of Artificial Organs*	To engineer longstanding adipose tissue without FGF2	*In vivo* animal model New Yealand white rabbits	Polypropylene column cage with pore size 200um Suspended type 1 collagen sponge	Synthetic and Biological Biodegradable	20 mm diameter 10 mm thickness 3.14 mL collagen suspension.	–	–
Debels *et al.*	Sustainable fat grafting. Optimizing fat grafting in an *in vivo* tissue engineering chamber model (Conference Abstract)	2013	*Wound repair and regeneration*	To improve long term outcome of fat grafting by adding a novel adipose derived matrix	*In vivo* animal model Rat model.	Adipose derived acellular matric (ADM)	Biological Biodegradable	2 mL	Minced autologous fat	Adipose dervied acellular matrix
Wang *et al.*	Combining decellularized human adipose tissue extracellular matrix and adipose-derived stem cells for adipose tissue engineering	2013	*Acta Biomaterialia*	To combine hDAM and ADSCs for adipose tissue engineering for soft tissue defect repair	*In vivo* animal model Nude rat model.	Decellularized human adipose tissue ECM (hDAM)	Biological Biodegradable	0.5 cm × 1 cm	Human adipose-derived stem cells	hDAM
Omidi *et al.*	Characterization and assessment of hyperelastic and elastic properties of decellularized human adipose tissues	2014	*Journal of Biomechanics*	To biomechanically characterise DAT scaffolds derived from various adipose depots in the body	*In vitro* model.	Decellularized adipose tissue (DAT)	Biological Biodegradable	Unknown	–	–
Debels *et al.*	Advances in Tissue Engineering; a Novel Technology Making Use of an *in vivo* Vascularized Chamber	2015	*Acta Chirurgica Belgica*	To engineer tissue using Adipogel and an arteriovenous loop within a TEC	*In vivo* animal model Rat model.	Adipose derived acellular matrix (ADM) (Adipogel) within a polycarbonate perforated hemispheric chamber.	Biological Biodegradable	2 mL	–	Adipose dervied acellular matrix
Giatsidis	Breast tissue engineering: Decellularized scaffolds derived from porcine mammary glands (Conference abstract)	2015	*Journal of the American College of Surgeons*	To investigate the effectiveness of decellularizing porcine mammary glands	*In vitro* model.	Decellularized porcine mammary glands	Biological Biodegradable	20 cm × 40 cm × 3 cm	–	–

**Table 2:** Synthetic scaffolds: systematic review data extraction

Authors	Title	Year	Journal	Study aim	Study model
Cho *et al.*	Engineering of volume-stable adipose tissues	2004	*Biomaterials*	To engineer adipose tissue using mechanical support structures	*In vivo* animal model Athymic mice.
Cho *et al.*	Engineered adipose tissue formation enhanced by basic fibroblast growth factor and a mechanically stable environment	2007	*Cell Transplantation*	To enhance adipose tissue regeneration by combining a mechanical support with bFGF	*In vivo* animal model Athymic mice.
Findlay *et al.*	Tissue-engineered breast reconstruction: bridging the gap toward large-volume tissue engineering in humans	2011	*Plastic and Reconstructive Surgery*	To engineer tissue together with a supportive vasculature in a large animal model	*In vivo* animal model Porcine.
Hettiarachichi *et al.*	The effects of biophysical and biochemical environment on preadipocyte differentiation (Conference abstract)	2012	*Journal of Tissue Engineering and Regenerative Medicine*	To investigate the effect of scaffold stiffness on adipocyte differentiation	*In vitro* model.
Shpaisman *et al.*	One-step synthesis of biodegradable curcumin-derived hydrogels as potential soft tissue fillers after breast cancer surgery	2012	*BioMacromolecules*	To develop a curcumin-derived hydrogel for use as a soft tissue filler and drug delivery system	*In vitro* model.
Chhaya *et al.*	Sustained regeneration of high-volume adipose tissue for breast reconstruction using computer aided design and biomanufacturing	2015	*Biomaterials*	To investigate PDLLA scaffolds for potential to engineer high volume adipose tissue	*In vivo* animal model Athymic nude rats.
Chhaya *et al.*	Transformation of Breast Reconstruction *via* Additive Biomanufacturing	2016	*Scientific Reports (Nature)*	To assess pre-vascularization and adipose tissue growth with a PCL scaffold	*In vivo* animal model Immunocompetent minipigs.
Morrison *et al.*	Creation of a Large Adipose Tissue Construct in Humans Using a Tissue-engineering Chamber: A Step Forward in the Clinical Application of Soft Tissue Engineering	2016	*EBioMedicine*	To engineer large clinically relevant volumes of adipose tissue in female patients with a TEC and fat flap	*In vivo* human case series Women with previous masectomies.
Wu *et al.*	Self-Assembling RADA16-I Peptide Hydrogel Scaffold Loaded with Tamoxifen for Breast Reconstruction	2017	*BioMed Research International*	To combine tamoxifen and a peptide scaffold for use as a soft tissue filler and drug delivery system	*In vivo* animal model Athymic mice.
Xu *et al.*	Self-assembling RADA16-I peptide hydrog elscaffold loaded with tamoxifen for breast reconstruction following lumpectomy (Conference abstract)	2017	*Clinical Therapeutics*	To combine tamoxifen and a peptide scaffold for use as a soft tissue filler and drug delivery system	*In vivo* animal model Athymic mice.
Xiao *et al.*	Pre-shaped large-volume engineered vascularized pedicled adipose flaps in a rabbit model: A two stage tissue engineering chamber-based procedure (Full Text not available)	2017	*Journal of Biomaterials and Tissue Engineering*	To engineer adipose tissue using a TEC and adipose flaps	*In vivo* animal model Rabbits.
Rossi *et al.*	Decoration of RGD-mimetic porous scaffolds with engineered and devitalized extracellular matrix for adipose tissue regeneration	2018	*Acta Biomaterialia*	To create a hybrid scaffold formed of a synthetic polymer and decellularized tissue	*In vivo* animal model Athymic mice.
O’Halloran *et al.*	Evaluating a novel adipose tissue engineering strategy for breast reconstruction post-mastectomy. (Conference Abstract)	2018	*Irish Journal of Medical Science*	To assess the oncological safety of ADSCs harvested from patients following chemotherapy	*In vitro* model
Gerges *et al.*	Exploring the potential of polyurethane-based soft foam as cell-free scaffold for soft tissue regeneration	2018	*Acta Biomaterialia*	To assess the biomechanical and physiochemical properties of a polyurethane-based scaffold and adipose tissue generation	*In vivo* animal model CD1 female mice
Leong *et al.*	ReFilx-synthetic biodegradable soft tissue fillers for breast conserving surgery in breast cancer (Conference abstract)	2018	*Cancer Research*	To evaluate ReFilx as a soft tissue filler for breast conserving surgery defects	*In vivo* animal model Yucatan minipigs.
Kaufman *et al.*	Interim report of a clinical registry: 669 patients implanted with a 3-d bioabsorbable marker (Conference abstract)	2018	*Annals of Surgical Oncology*	Interim report to summarize data collected in an IRB-approved Registry	*In vivo* human case series.

Authors	Scaffold	Scaffold—biological/synthetic, bio/non-biodegradable	Volume/size of scaffold	Cell therapy	Cell niche ± growth factor
Cho *et al.*	Dome shaped support structure formed of Poly(glycolic acid) (PGA) and Poly(L-lactic acid) (PLLA).	Synthetic Biodegradable	0.12 cm^3^	Human Preadipocytes	Thrombin—fibrinogen solution with heparin (forming a fibrin gel) Basic fibroblast growth factor (bFGF).
Cho *et al.*	Dome shaped support structure formed of Poly(glycolic acid) (PGA) and Poly(L-lactic acid) (PLLA).	Synthetic Biodegradable	0.12 cm^3^	Human Preadipocytes	Thrombin—fibrinogen solution with heparin (forming a fibrin gel) Basic fibroblast growth factor (bFGF).
Findlay *et al.*	Perforated polycarbonate chamber with a poly(L-lactide-co-glycolide) sponge in half the chambers.	Synthetic Biodegradable	78.5 mL	5 mL adipose tissue flap	–
Hettiarachichi *et al.*	Polyacrylamide gel	Synthetic Biodegradable	Unknown	3T3-L1 cells	Normal or adipogenic media
Shpaisman *et al.*	Curcumin-derived hydrogel. (Nontoxic Poly(ethylene glycol) and desaminotyrosyl-tyrosine ethyl ester)	Synthetic Biodegradable	Unknown -Hydrogel	–	–
Chhaya *et al.*	Poly(D,L)-Lactide polymer (PDLLA).	Synthetic Biodegradable	3 cm^3^	Human umbilical cord perivascular cells for 6 weeks. Then human umbilical vein endothelial cells (HUVECs).	Thrombin—fibrinogen solution.
Chhaya *et al.*	Medical grade polycaprolactone (mPCL).	Synthetic Biodegradable	75 cm^3^	Autologous lipoaspirate	–
Morrison *et al.*	Dome shaped, hollow, 3 mm thick perforated acrylic chambers.	Synthetic Non-biodegradable	140-360 mL (custom made)	Thoracodorsal artery perforator (TAP) fat flap.	–
Wu *et al.*	Tamoxifen loaded—RADA16-I peptide hydrogel	Synthetic Biodegradable	Unknown—Hydrogel	Human adipose-derived stem cells (hADSCs)	High-glucose DMEM supplemented with 10% FBS, dexamethasone, isobutylmethylxanthine, insulin, indomethacin.
Xu *et al.*	Tamoxifen loaded—RADA16-I peptide hydrogel	Synthetic Biodegradable	Unknown—Hydrogel	Human adipose-derived stem cells (hADSCs)	–
Xiao *et al.*	Tissue Engineering Chamber (TEC); unknown material	Synthetic Non-biodegradable	Unknown	Adipose flaps (0.8 mL) with vessel pedicle	–
Rossi *et al.*	Hybrid ECM-OPAAF scaffold ECM from decellularized hASCs OPAAF—RGD-mimetic poly(amidoamine) oligomer macroporous foam.	Synthetic and biological Biodegradable	8 mm diameter 3 mm thickness.	–	Initially FGF-2, insulin, dexamethasone, indomethacin, IBMX prior to decellularization
O’Halloran *et al.*	Hydrogels of 2% w/v hyaluronic acid (2× crosslinking density loaded with adipocytes at 6.7% total gel volume)	Synthetic Biodegradable	Unknown—Hydrogel	Adipose Derived Stem Cells (ADSCs)	–
Gerges *et al.*	Poly (urethane)-based scaffolds	Synthetic Biodegradable	8 mm diameter 4 mm thickness.	–	–
Leong *et al.*	Polyurethanes (amino-acid based)	Synthetic Biodegradable	Unknown—hydrogel	–	–
Kaufman *et al.*	3-D bioabsorbable implant (unknown material)	Unknown Biodegradable	Unknown	–	–

**Table 3:** Nipple areolar complex scaffolds: systematic review data extraction

Authors	Title	Year	Journal	Study aim	Study model	Scaffold	Scaffold—biological/synthetic, bio/non-biodegradable	Cell therapy	Cell niche ± growth factor
Cao *et al.*	Tissue-engineered nipple reconstruction	1998	*Plastic and Reconstructive Surgery*	To tissue engineer autologous cartilage in the shape of a human nipple	*In vivo* animal model Porcine.	Pluronic F-127 hydrogel (polyethylene oxide and polypropylene oxide copolymer).	Synthetic Non-biodegradable (Scaffold dissolves over time).	Autologous chrondrocytes.	–
Tierney *et al.*	Biologic collagen cylinder with skate flap technique for nipple reconstruction	2014	*Plastic Surgery International*	To reconstruct the nipple using a biologic collagen cylinder with skate flap	*In vivo* human case series.	Rolled cylinder of ECM collagen derived from porcine small intestinal submucosa	Biological Biodegradable	–	–
Pashos *et al.*	A tissue engineered nipple and areola complex (Conference abstract)	2015	*Molecular Therapy*	To design a scaffold formed of a decellularized whole NAC for tissue engineering	*In vivo* animal model Rhesus Macaque Non-human primates.	Decellularized NAC	Biological Biodegradable	Rhesus bone marrow-derived stem cells	–
Pashos *et al.*	Characterization of an acellular scaffold for a tissue engineering approach to the nipple–areolar complex reconstruction	2017	*Cells Tissues Organs*	To create a nonimmunogenic scaffold from a decellularized NAC for use as an onlay graft that is patient specific	*In vivo* animal model Rhesus Macaque Non-human primates.	Decellularized NAC	Biological Biodegradable	Bone marrow-derived mesenchymal stem cells (BMSCs) from rhesus macaques	a-modified Eagles medium (fetal bovine serum, l-glutamine, penicillin, streptomycin, amphotericin

## Discussion

### Scaffolds

Since the idea of a tissue engineered breast was first proposed, researchers have been analysing materials suitable to use as an implantable scaffold that would support growth and allow regeneration of host tissue. An ideal scaffold would combine the key principles of biodegradability, low immunogenicity, porous architecture and structural integrity. The scaffold composites can broadly be categorized into biological scaffolds and synthetic scaffolds.

#### Biological Scaffolds

Eight articles were identified that utilized a biological scaffold. All were biodegradable and could be sub-divided into two techniques; either a collagen based scaffold as a gel or sponge, or a decellularized tissue based scaffold created from adipose tissue, rat lungs or porcine mammary glands.

#### Collagen-Based

Huss and Kratz published the first step toward regenerating human autologous breast tissue on a 3-D biological matrix.^22^ Human mammary epithelial cells with adipose tissue were cultured *in vitro* on collagen gel. The typical growth pattern comprised large epithelial patches of fibroblast-like shaped cells with preadipocytes in between acquiring a round shape and accumulating lipids with time. Cells require adhesive materials in order to survive and type I collagen is known to have an excellent porous structure; a suitable scaffold for cell migration and proliferation.^20^ Utilizing a polypropylene cage implanted into the bilateral fat pads of rabbits and injecting a 3.14 mL suspension of minced type I collagen sponge and saline, a study reported significant adipogenesis filling 92.8 ± 6.6% of the cage volume after 12 months.^42^ Significant volumes of adipose tissue had been generated from surrounding tissue, along with the endogenous production of growth factors essential for adipogenesis and angiogenesis. These included fibroblast growth factor-2 (FGF2), vascular endothelial growth factor (VEGF), platelet-derived growth factor (PDGF), epidermal growth factor (EGF) and insulin-like growth factor-1 (IGF-1), that were all detectable in the wound drainage fluid. Although the polypropylene cage used was non-absorbable and too hard for breast reconstruction, continuing the search for the appropriate scaffold.

#### Decellularized Tissue

The search began to look at the concept of decellularizing tissue; using a solution to completely remove the cellular component leaving only the extracellular matrix (ECM) to form a scaffold. Tada and Fujisato^40^ showed that rat lungs could be decellularized, injected with adipocytes and implanted into nude mice. Several researchers developed protocols for decellularizing adipose tissue^11^,^32^,^40^,^43^; decellularized adipose tissue (DAT), decellularized human adipose tissue ECM (hDAM) or adipose derived acellular matrix (ADM). To aid adipocyte infiltration and proliferation into the scaffold, and overall structure of the reconstructed breast, it is crucial that the scaffold have similar biomechanical properties and deformability to that of native breast tissue. Omidi *et al.*^32^ showed that DAT scaffolds sourced from different areas of female patients (breast, subcutaneous abdominal region, omentum, pericardial depot or thymic remnant) all exhibited linear elastic and hyperelastic properties alike, and were consistent with the previously reported Young’s modulus of adipose breast tissue^36^; an average value of 3250 ± 910 Pa. Advantageously this would mean the DAT scaffolds could be sourced from any of the areas for commercial use and would demonstrate similar stiffness and deformability as a natural breast under gravity loading from prone to supine body positions.

**Figure 2:**
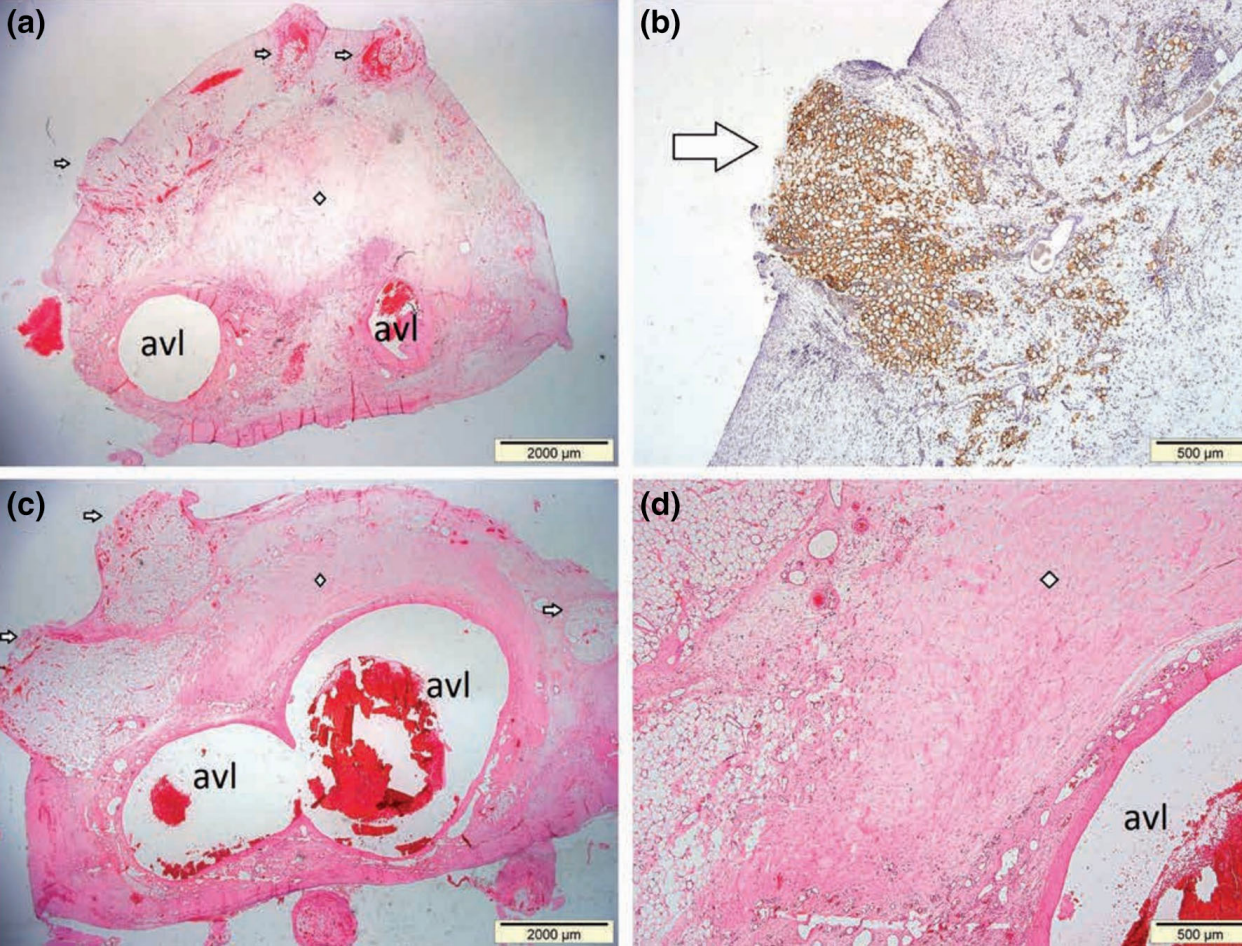
H&E stained cross sections demonstrating tissue scaffold. (a, b) At 6 weeks. avl = arteriovenous loop. The arrows demonstrate areas of tissue growth within the nubbins of the TEC and the diamond areas of un-remodeled Adipogel. (b) the brown staining demonstrating viable fat cells. (c, d) exhibit scaffold at 12 weeks with substantially more viable adipose tissue. *Source* Debels^11^

Subcutaneously implanting the hDAM scaffolds (0.5 cm × 1 cm) in a nude rat model exhibited a minimal inflammatory response after 30 days without obvious rejection, with initially well vascularized grafts.^43^ However from week 2 to 8, there was a significant decrease in graft vascularization; this was thought due to a gap still present between the scaffold and native adipose tissue. To improve graft vascularization, a femoral arteriovenous loop was trialled inside a perforated tissue engineering chamber (TEC; formed of polycarbonate) containing 2 mL of ADM termed “Adipogel” using a rat animal model.^11^ At both 6 and 12 weeks post implantation, the chambers were half filled with well vascularized connective tissue (0.946 mL ± 0.161), but only containing 135 *µ*L ± 76 adipocytes along with some myocytes. The adipose tissue appeared to be away from the arteriovenous loop at the outer margin of the TEC, while muscle cell growth was at the arteriovenous loop origin near the inguinal-abdominal muscle intersection (Fig. 2). Debels hypothesized this is either due to migrating stem cells or cell-to-cell signaling pathways from local native tissue. Meanwhile all chambers demonstrated an immunological reaction and were encased by an external fibrotic capsule. Giatsidis^19^ took the decellularization concept further, applying the technique to porcine mammary glands. The resultant scaffold preserved original morphology with histological analysis resembling native architecture of ECM and vascular/ductal networks. This study also demonstrated the benefit of harvesting adjacent glands together and molding the scaffold to the required shape.

The major limitations of biological scaffolds include rapid enzymatic and hydrolytic degradation, along with a strong immunogenic response *in vivo*. Techniques have been developed to decrease the rate of degradation, such as enzymatic pre-treatment or cross-linking using various agents, which improve robustness and maintain integrity; however the effectiveness can vary.^30^ A key requirement of the scaffold is to provide mechanical support during regeneration until the tissue is mature enough to support itself, which unfortunately biological materials have been unable to guarantee. Hence current research is now focussing on utilizing synthetic polymers.

#### Synthetic Scaffolds

Fifteen articles were identified that utilized synthetic scaffolds, with 13 different techniques. These comprised of eleven biodegradable scaffolds and two non-biodegradable tissue engineering chambers (TEC) that are to be removed once the tissue has generated.

The majority of these scaffolds are synthesized from thermoplastic polymers and can be further sub-divided depending on their structure; either a hydrogel filler or a solid structural support.

#### Hydrogel Structure

As with biological scaffolds, the biomechanical and biochemical environment of synthetic scaffolds influences adipocyte migration and proliferation. Hettiarachichi *et al.*^21^ were investigating the stiffness of a polyacrylamide gel and effect on adipocyte differentiation *in vitro*. The most favourable matrices were those that had a similar stiffness to endogenous adipose tissue; Young’s moduli above 4.1 kPa elicited maximal spreading of adipose cells, while moduli below 4.1 kPa a more spherical phenotype.

Along with providing a soft tissue support to replace breast tissue, the idea that hydrogels could be used as drug delivery systems was hypothesized. Curcumin-derived hydrogels were synthesized from poly(ethylene glycol) (PEG) and desaminotyrosyl-tyrosine ethyl ester (DTE), that upon hydrogel degradation led to local release of active curcumin (CUR).^37^ Through a condensation polymerization protocol, different compositions of hydrogel could be synthesized that altered its overall properties, including curcumin concentration and swelling ability; CUR_50_PEG_50_ exhibited the most favourable with stable curcumin release and compression modulus comparable with native breast tissue. *In vitro* analysis demonstrated selective cytotoxicity against breast cancer cells, but no cytotoxicity to noncancerous primary human dermal fibroblasts, further suggesting promising use as a bioactive void filler for excised cancerous tissue.

Additional attempts to engineer artificial therapeutic breast tissue led to the efficacious incorporation of tamoxifen into a self-assembled injectable polypeptide, RADA16-I.^44^ The hydrogel scaffold provided support for cell attachment and proliferation, with results suggesting the 3D environment enhanced toxicity of tamoxifen on breast cancer cells, while reducing the effect on human adipose derived stem cells (hADSCs). After subcutaneous implantation into nude mouse animal models, the scaffold formed a round mass with regular shape and clear edge, retaining its shape upon compression. Although the scaffold was completely degraded and absorbed within 7 days *in vivo*, highlighting long-term persistence as the major challenge. For the time being, therapeutic benefits of bioactive implants in human based studies are limited in oncoplastic surgery, only providing tissue support and a tumor bed target for radiation therapy.^25^

Poly(urethane)-based scaffolds have shown promise for use as hydrogel fillers to restore breast volume.^18^,^27^ A polyurethane-based soft foam (PUF) demonstrated fatigue resistance and tuneable mechanical properties by adjusting the ratio of poly(ethylene glycol) (PEG) to polyester (PE) segments. The optimum balance shown to be PUF 3/10 (PEG/PE) enhanced the hydrophilic character of the scaffold, favouring efficacious diffusion of body fluids. Although degradation kinetics showed after 6 months loss of more than 50% original weight. *In vivo* the undifferentiated mesenchymal cells attached themselves to the periphery of the scaffold, gradually infiltrating toward the centre. By day 91, initial loose fibromyxoid tissue had been partially replaced by mature adipose tissue. Expectedly the scaffold elicited a foreign body reaction, although only a partial fibrous capsule was noted up to 3 months.

#### Solid Structure

Several solid dome shaped support structures synthesized from polymers have been analysed. Cho *et al.*^9^,^10^ indicated that a mechanically stable environment is crucial to maintain engineered tissue volume, creating a poly(glycolic acid) matrix (PGA) coated in poly(l-lactic acid) (PLLA). The structure was formed by molding strips of PGA mesh and then immersing in PLLA, and was able to withstand compressive forces, maintaining stable *in vivo* volume (0.12 cm^3^) at 6 weeks. Although the domed structure was hollow, minimizing surface area for cell attachment which would make engineering large volumes of tissue difficult.

A 3D printer has been utilized to design and create a poly(d,l)-lactide polymer (PDLLA) scaffold,^8^ allowing the ability to customise size and shape of the engineered breast. Internal morphology of the scaffold, including porosity and pore size, can be tailored to individual patients. The PDLLA scaffold (volume 3 cm^3^) withstood contraction forces for at least 6 months *in vivo* (nude rat model) and did not exhibited any mass loss. Pore sizes of 1.5 mm allowed for tissue and vascular ingrowth, with 81% of the overall tissue at 24 weeks composed of adipose tissue; the majority being host-derived adipocytes. Scaffolds were associated with minimal inflammatory reaction, integrating into the host body and surrounded by a fibrotic capsule that decreased as the scaffold degraded.

**Figure 3:**
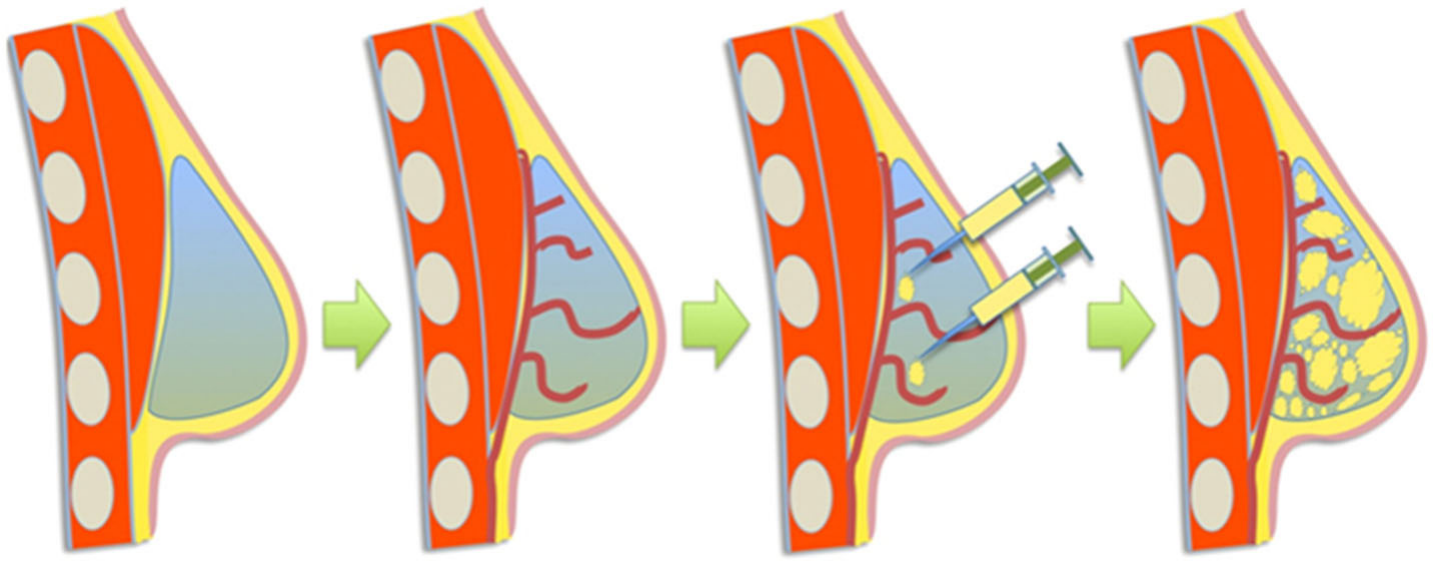
“Overall concept of the prevascularization and delayed fat injection concept.” An empty scaffold is implanted onto the chest wall, and after a period of prevascularization, lipoaspirate is injected into the construct. *Source*: Chhaya *et al.*^7^

Chhaya *et al.*^7^ went on to develop another custom-made scaffold (volume 75 cm^3^), using medical-grade polycaprolactone (mPCL) through additive biomanufacturing. On the contrary to previous designs, the slowly degrading mPCL scaffold had a stiffness value three orders of magnitude higher than that of native breast tissue, hypothesizing that newly regenerated adipose tissue needed protection from compressive and shear forces until it had matured. Scaffolds were implanted into immunocompetent minipigs and allowed a period of prevascularization before cellular addition (Fig. 3). Prevascularization allowed a platelet-rich fibrin blood clot to form inside the large pore network, stimulating angiogenesis and the production of a well vascularized connective tissue. It was hypothesized that altering the initial scaffold treatment protocol (empty scaffold vs. prevascularization plus cellular addition) could modify the composition of connective tissue to adipose tissue, tailoring the procedure for aesthetic augmentation or breast reconstruction. Upon explantation there were no major signs of inflammation within the tissue section or scaffolds, however there were low-grade granulomatous reactions localized around the scaffold. The role of macrophages is not fully understood, however Chhaya *et al.* stipulate it could contribute to angiogenesis, with prior research potentially linking the two *via* the secretion of vascular endothelial growth factor (VEGF) and platelet derived growth factor (PDGF).^39^

**Figure 4:**
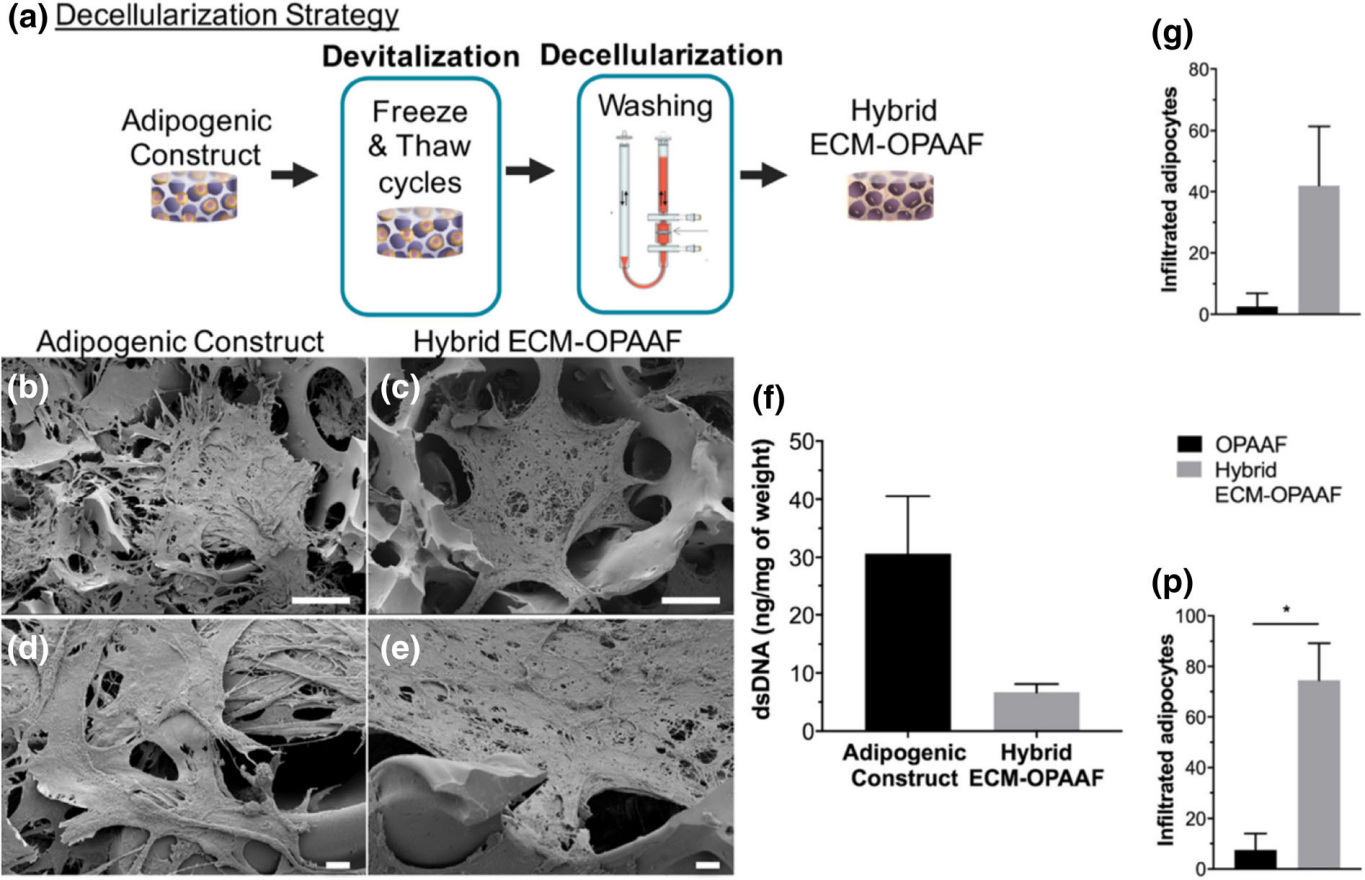
(a) The decellularization protocol. (b–e) The scanning electron microscope (SEM) analysis of the constructs (scale bars: 100 *µ*m for (b and c) 10 *µ*m for (d and e)). (f) Successful decellularization with a reduction in DNA content. (g, p) The adipocyte infiltration at 2 weeks and 4 weeks post implantation respectively. The hybrid ECM-OPAAF construct shows superior adipoinductive capacity. *Source* Rossi *et al.*^35^

The major disadvantage of synthetic scaffolds compared to biological scaffolds when seeding with cell and tissue components is the lower cellular affinity to the synthetic material. To improve this, Rossi *et al.*^35^ has attempted to combine the functionalities of a synthetic polymer with a biological matrix to engineer a hybrid scaffold; although this would increase the cost and complexity of the biomaterial. A RGD-mimetic poly(amidoamine) oligomer microporous foam (OPAAF) was created by free radical polymerization. The hydrophilic 3D interconnected porous network was decorated and then decellularized to generate a hybrid adipose ECM-OPAAF construct (Fig. 4a). The study describes an effective decellularization protocol that maintains ECM architecture. While the original OPAAF scaffold had similar mechanical properties to that of native adipose tissue, the hybrid ECM-OPAAF construct had a Young’s modulus of more than double; 10.5 ± 3.4 kPa. *In vitro* and *in vivo* analysis demonstrated a strongly enhanced adipoinductive capacity as a result of ECM decoration, with an increase of infiltrating native adipocytes (Figs. 4g and 4p). The interfacial biocompatibility between host and scaffold was difficult to evaluate in the immunocompetent mouse model, with a strong immune response against the human ECM. Although *in vitro* co-culture with human peripheral blood exhibited a more pro-regenerative macrophage response.

As with biological scaffolds, vascularization of a large volume of regenerated tissue in synthetic scaffolds has been one of the main limiting variables. Utilizing previous knowledge, a small experimental pig animal study focussed on the co-development of adipose tissue alongside a supportive vasculature.^14^ Pedicled fat flaps based on superficial circumflex iliac vessels were inserted into tissue engineering chambers (TEC; perforated polycarbonate) together with a poly(L-lactide-co-glycolide) (PLGA) sponge (volume 78.5 mL). At 22 weeks (12 weeks after chamber removal) the initial 5 mL adipose tissue flaps had expanded into a larger core of 56.5 mL adipose tissue surrounded by a fibrous capsular rim, with growth associated with adipocyte hyperplasia. One construct was even transferred and survived on its pedicle in an adjacent submammary pocket. Even though the study has a small sample and no control group to compare the PLGA sponge, it provides proof of principle the technique can be used on a larger scale.

The concept was further demonstrated in a rabbit animal model.^45^ An 8 mL volume TEC was implanted subcutaneously with a vessel pedicled 0.8 mL adipose flap inserted. The flaps expanded containing adipose tissue within and after TEC removal at 8 weeks, growth continued until stabilization at weeks 12–24.

**Figure 5:**
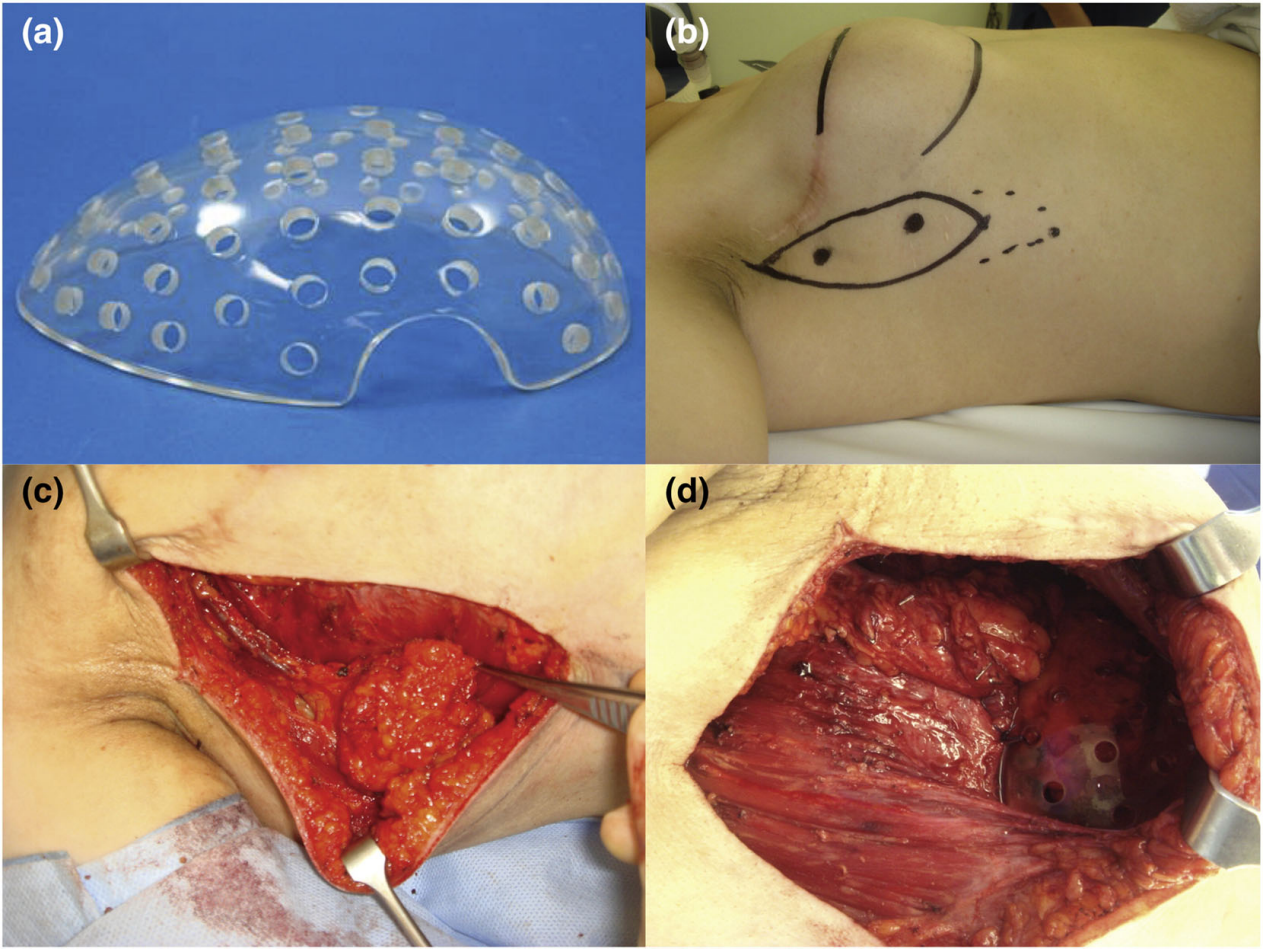
(a) demonstrates the 210 cm^3^ TEC. (b–d) The TAP fat flap design, the surgical procedure and the final result prior to closing. *Source* Morrison *et al.*^29^

**Figure 6:**
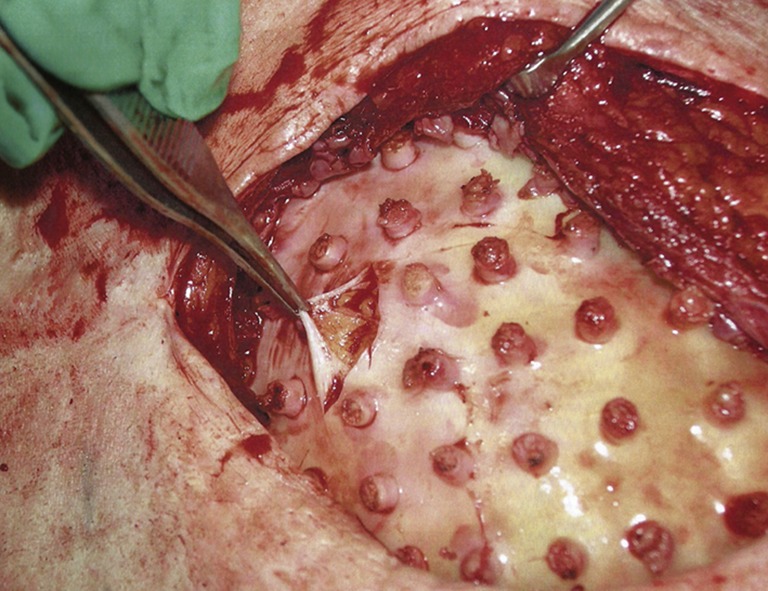
At 12 months, the tissue generated exhibits adipose tissue covered by a fibrous capsule. *Source* Morrison *et al.*^29^

The proof of concept led to the creation of a large volume of adipose tissue construct in humans.^29^ A study of five women who had had previous mastectomies incorporated a thoracodorsal artery perforator (TAP) fat flap within a TEC (perforated acrylic) (volume 140–360 mL) under a submuscular plane (Fig. 5). At 6 months follow-up the TECs were removed. While only successful in one patient, 210 mL of newly formed tissue with “macroscopic appearance and palpable texture very similar to native adipose tissue” (Fig. 6) provides evidence of the feasibility of a tissue engineering approach in humans.

### Nipple–Areolar Complex

Even though the focus of this systematic review is on reconstructing the breast, the literature search returned four articles investigating the nipple-areolar complex (NAC) and it was deemed imperative to mention. Thankfully nipple-sparing mastectomy is becoming increasingly more common however novel tissue engineering reconstructive techniques must be compatible with both nipple-sparing and non-nipple-sparing techniques. The scaffold composites included one collagen based, two decellularized tissue based and one thermoplastic polymer based.

Previously breast reconstruction had been aimed at improving cosmesis and symmetry of female form with clothes on. Eventually more attention was attributed to the NAC to improve naked female form and skin flaps were constructed that could also be tattooed for pigmentation.^38^

As with the breast, a tissue engineering approach has been proposed. Cao *et al.*^5^ described a simple technique by injecting a pluronic F-127 hydrogel seeded with autologous chondrocytes into a swine animal model. Surrounded by a purse string suture led to the generation of stable cartilage tissue that at 10 weeks closely resembled a NAC.

Additional attempts now include a rolled cylinder of collagen derived from the submucosa of porcine small intestine being used in conjunction with a skate flap in a human based study.^41^ The outcome at the time of surgery ranged from 6 to 7 mm nipple projection, with a 30–50% loss at 6 months.

Alternatively a decellularized NAC seeded with autologous cells to be used as an onlay graft is in initial stages of testing.^33^ The *in vitro* report demonstrates preservation of the NAC primary structures on a microscopic scale with an effective decellularization protocol. Reseeding of the scaffolds with bone marrow-derived mesenchymal stem cells exhibited initial attachment to the periphery, with cells migrating deeper into the scaffold by day 7; 91% cell viability at 48 h and 67% undergoing proliferation by day 7.

### Cellular Therapy and Growth Factors

The idea is to regenerate host tissue to replace excised breast tissue and provide soft tissue support. So far different materials and properties of scaffolds have been characterized. The scaffold needs to provide structural integrity until regenerated tissue has matured to support itself, upon which the scaffold degrades (or is removed) leaving only host tissue. There are two main hypothesises to regenerate tissue within scaffolds. Either scaffolds provide the space and environment for surrounding native cells to migrate into the scaffold and proliferate or cells, ideally autologous to prevent immunogenicity, are implanted into scaffolds and proliferate.

Fifteen of the aforementioned articles investigated incorporation of cellular therapy to improve adipogenesis, including bone marrow derived stem cells, adipose derived stem cells (ADSCs), preadipocytes and lipoaspirate. Along with this, eight included use of culture medium containing growth factors and proteins. While only one article evaluated the effect of a specific growth factor and compared this with a control group.

Chhaya *et al.*^7^ hypothesized that initial scaffold treatment strategy could be tailored according to the indication for the surgical procedure. An empty scaffold for post-mastectomy breast reconstruction would allow native cells to migrate in and generate organized connective tissue, avoiding risk of breast cancer recurrence from using adipose progenitor cells. While implanting autologous lipoaspirate into scaffolds for aesthetic breast augmentation would allow generation of higher composition of adipose tissue to connective tissue, 47.32% ± 4.12, maintaining natural tactile sensation and mimicking morphology of the breast.

Others have likewise discovered that implanting autologous lipoaspirate into the scaffold resulted in the significant increase of viable adipose tissue^12^; although the majority of originally inserted adipocytes had died, indicating neoadipogenesis. This can occur due to the presence of adipose derived stem cells (ADSCs) within lipoaspirate, being first isolated and reported by Zuk *et al.*^46^ ADSCs are multipotent with potential to differentiate into adipose tissue, osteocytes, chondrocytes or myocytes when cultured in the presence of specific differentiation factors. To direct ADSCs toward the adipose lineage *in vitro*, they can be cultured in high-glucose Dulbecco’s modified Eagle’s medium (DMEM) supplemented with 10% fetal bovine serum, dexamethasone, isobutylmethylxanthine, insulin and indomethacin.^44^ Though there is potential risk of implanted ADSCs stimulating breast cancer recurrence, ADSCs have been isolated from breast tissue and abdomen of patients treated with neoadjuvant chemotherapy and demonstrated better adipogenic potential and improved oncological safety with decreased expression of cancer driver genes.^31^

Once implanted, a trial studied the effect of a fibrin gel containing basic fibroblast growth factor (bFGF), concluding there was a significant increase in preadipocyte cell survival, neovascularization and volume of generated adipose tissue when compared to a control group.^9^ Cho *et al.* hypotheses two mechanisms for enhanced adipogenesis. Firstly that neovascularization in fibrin gel may be enhanced by bFGF, leading to factors secreted from vasculature endothelial cells that are known to promote adipocyte precursors to migrate and proliferate.^1^,^16^ Secondly that bFGF may directly stimulate implanted preadipocytes to differentiate into adipocytes.

Other factors effecting cell migration and proliferation include the environment surrounding the cells, termed the cell niche. The hybrid ECM-OPAAF scaffold characterized previously^35^ demonstrated superior adipoinductive capacity and it was hypothesized this maybe due to the presence of adipokines embedded within the ECM that were deposited by ADSCs prior to decellularization. Cell signaling, whether in the form of cytokines, adhesion molecules or cell-to-cell contact, is thought to be imperative in tissue engineering. Immunohistochemical (IHC) analysis of the hDAM scaffold developed by Wang *et al.*^43^ reported a well maintained composition of collagen, glycosaminoglycan and VEGF; the latter stimulating angiogenesis and neovascularization. Although the scaffold lacked laminin, important in cell attachment and adhesion. Implanting ADSCs into the scaffold *in vivo*, the density of donor cells was preserved up to week 4, but then decreased significantly to week 8. On the contrary, the density of host native cells significantly increased from week 4 to 8, indicating host tissue integration and adipose tissue regeneration. What caused the initial survival and then loss of ADSCs? What caused the subsequent migration of host cells after 4 weeks of implantation? There remains a substantial unknown knowledge relating to the interaction between scaffold, cellular addition and growth factors.

Lastly, to improve vascularization a study trialled inserting human umbilical cord perivascular cells (HUCPVCs) within fibrin gels containing heparin into PDLLA scaffolds for 6 weeks *in vitro* and then upon implantation into rat animal models, human umbilical vein endothelial cells (HUVECs) were injected.^8^ MicroCT angiography reported a high degree of vascularization equally distributed throughout scaffolds, and HUVECs survived the 6 month implantation process, but self-assembled functional capillary networks were only observed around the periphery of the scaffolds.

### Vascularization of Engineered Tissue

The optimal vascularization of the newly regenerated tissue has proved a major challenge and although already discussed in part previously, its importance should be highlighted further. Scaffolds may show adequate properties with *in vitro* and small animal models, although upon upscaling to larger animals or even the female patient, issues can arise with insufficient vascularization of central zones leading to tissue necrosis. The most notable techniques identified in this review were the pre-vascularization concept and the inclusion of an arterio-venous loop.

Chhaya *et al.*^7^ hypothesized a novel pre-vascularization concept whereby a scaffold is implanted and allowed a period of 14 days prior to delayed fat injection. This period allowed the formation of a blood clot within the scaffold’s interconnected porous network, consisting of platelets embedded within cross-linked fibrin fibres and the endogenous production of growth factors including fibronectin, vitronectin and thrombospondin. The authors correlate this process to previous literature, indicating the stimulation of a strong angiogenic response and the formation of highly organized connective tissue. Following the 14 day period, delayed fat injection provided an adipogenic stimulus that lead to the engineering of tissue filling the entire 75 cm^3^ scaffold, with the composition of 47.32% adipose tissue. The initial connective tissue had been modified to contain highly vascular areas of fat tissue and no evidence of tissue necrosis over the 24-week study period. To further the concept, it will need to be studied utilizing a larger scaffold to assess the feasibility of engineering clinically relevant volumes of adipose tissue.

Several articles evaluated the use of an arterio-venous loop and the most successful to date has been reported in a human case series. Morrison *et al.*^29^ implanted a custom-made TEC in a submuscular pocket on the chest wall of female patients with prior mastectomy. A thoracodorsal artery perforator fat flap was transposed into the TECs and upon TEC removal 12 months later, 210 mL of tissue with macroscopic appearance of fat had been engineered that bled when punctured, indicating good vascularization. The mechanism is not fully understood, but hypothesized to be hypertrophy and hyperplasia, expanding the existing tissue of the transposed fat flap. By stretching of the overlying tissue and creating a non-collapsible space with the TEC, ischemia is likely to occur and therefore enhance angiogenesis and the sprouting of blood vessels from the vascular pedicle.

### Study Limitations

The systematic review had several limitations. As the concept of a tissue engineered breast is still in its infancy, the authors’ protocols and techniques were diverse with no standardization. Along with relatively small sample sizes and low study powers, made any form of comparison challenging. Lastly, 10 of the articles were conference abstracts with limited information and the full text of one article was unavailable.

## Conclusion and Future Work

To conclude, current research in tissue engineering is demonstrating promising results for the future of breast reconstruction. The most popular strategy focusses on regenerating breast tissue using a biodegradable synthetic scaffold to support cell migration and proliferation.

Exploring the literature has identified keys questions requiring further research, which can be divided into three areas. Firstly the scaffold itself. The internal morphology requires optimising to aid cell migration and improve vascularization; the latter limiting the volume of tissue generated. Together with what amount of time is needed for the mature regenerated tissue to be able to support itself, at which point the scaffold should degrade. This leads onto question the long-term safety of the scaffolds with polymer degradation products unknown. Secondly, the addition of cells. Will the use of stem cells or lipoaspirate improve adipogenesis or is it safer to allow native tissue to migrate avoiding the oncogenesis risk? And lastly, what are the effects of specific growth factors on both the tissue generated and the development of well-organized vasculature?
